# Liver and spleen ultrasonography and elastography are useful for identifying a history of upper gastrointestinal bleeding in patients with schistosomiasis

**DOI:** 10.1186/s12879-025-11635-6

**Published:** 2026-01-29

**Authors:** Caroline Louise Diniz Pereira, Ana Lúcia Coutinho Domingues, Joelma Carvalho Santos, Iris Campos Lucas, Eduardo Sampaio Siqueira, Carlos Alexandre Antunes de Brito, Edmundo Pessoa Lopes

**Affiliations:** 1 Postgraduate Program in Tropical Medicine, Center of Medical Sciences, Universidade Federal de Pernambuco (UFPE), Recife, Pernambuco 50670-901 Brazil; 2 Department of Internal Medicine, Gastroenterology Division, Center of Medical Sciences, Universidade Federal de Pernambuco (UFPE), Recife, Pernambuco 50670-901 Brazil; 3 Immunohematology Laboratory, Hemocentro de Alagoas (Hemoal), Maceió, Alagoas 57010-382 Brazil; 4 Endoscopy Service, Hospital das Clínicas/EBSERH, Universidade Federal de Pernambuco (UFPE), Recife, Pernambuco 50670-901 Brazil; 5 Department of Immunology, Autoimune Research Institute, Recife, Pernambuco 52011-040 Brazil; 6 Center of Medical Sciences, Universidade Federal de Pernambuco (UFPE), Av. Prof. Moraes Rego, 1235 – Cidade Universitária, Recife, PE 50670-901 Brazil

**Keywords:** Spleen stiffness, Liver stiffness, Portal hypertension, Elastography, Non-invasive diagnostics

## Abstract

**Background:**

*Schistosoma mansoni* infection can lead to periportal fibrosis (PPF), non-cirrhotic (pre-sinusoidal) portal hypertension, development of varices, and gastrointestinal bleeding. In this context, we evaluated hepatic disease morbidity and signs of portal hypertension in patients with schistosomiasis, with and without a history of upper gastrointestinal bleeding (UGIB), using ultrasonography and point shear wave elastography (pSWE) of the liver and spleen.

**Methods:**

An analytical case-control study involving 177 patients with schistosomiasis, with and without a UGIB history, conducted at the Gastroenterology Division of the Hospital das Clínicas-UFPE, between 2018 and 2024. All patients underwent upper abdominal ultrasonography and hepatic and splenic stiffness on pSWE.

**Results:**

Among the 177 patients with schistosomiasis, 91 (51.4%) were women; with a median age of 55 years; 51 patients (28.8%) reported a history of UGIB. These 51 patients presented more advanced PPF patterns, larger portal and splenic vein diameters, increased longitudinal and transverse spleen diameters (splenic index), and greater liver and spleen stiffness on pSWE. Most of them (84.3%) had advanced PPF (patterns E or F), which are associated with marked splenic enlargement and an increased risk of portal hypertension. The ROC curve analysis identified the following cut-off values for distinguishing patients with a history of UGIB: portal vein diameter > 1.3 cm, splenic vein diameter > 0.79 cm, splenic index > 65.2, liver and spleen pSWE velocity > 1.5 m/s and > 3.56 m/s, respectively. Among these parameters, the splenic index demonstrated the highest high accuracy (AUC = 0.804) in identifying patients with a history of UGIB and a robust performance in ruling out this condition with a negative likelihood ratio (LR-) of 0.064. This finding implies that a splenic index < 65.2 is a strong predictor of the absence of UGIB.

**Conclusion:**

Ultrasonographic and elastographic parameters, particularly those related to the spleen, with emphasis on the splenic index, proved to be promising tools for identifying patients with schistosomiasis with a history of UGIB.

**Ethics Statement:**

The study was approved by the Research Ethics Committee of the Health Sciences Center at Universidade Federal de Pernambuco (UFPE) (Approval No. 7.112.760/2024), and all participants provided written informed consent. The study was conducted in full accordance with the ethical principles outlined in the Declaration of Helsinki of the World Medical Association.

**Supplementary Information:**

The online version contains supplementary material available at 10.1186/s12879-025-11635-6.

## Background

The deposition of *Schistosoma mansoni* eggs in portal vein terminal branches induces a granulomatous response, leading to the formation of periportal fibrosis (PPF) and progressive obstruction of blood flow within the liver, resulting in pre-sinusoidal non-cirrhotic portal hypertension [1, 2]. Simultaneously with this obstruction, splenomegaly develops due to the immunological stimulation triggered by worms and eggs, along with increased splenoportal blood flow, giving rise to the hepatosplenic schistosomiasis (HSS) form [3]. It is therefore assumed that non-cirrhotic portal hypertension in HSS results from both the obstructive process and hyperflow in the splenic vein, which leads to the development of collateral vessels and varices [2, 3]. Varices can cause upper gastrointestinal bleeding (UGIB) in 20–40% of cases with HSS form [4, 5].

Thus, patients with HSS may undergo upper gastrointestinal endoscopy to assess the risk of bleeding [6, 7]. It is noteworthy that gastrointestinal bleeding represents a milestone in the natural history of HSS, with devastating consequences [7]. Following recurrent bleeding due to portal hypertension, hepatocyte ischemia often occurs, leading to hepatic function decompensation and increased susceptibility to infections, hypoalbuminemia, ascites or acute kidney injury, what may significantly raise the patient’s risk of death [8–12].

Ultrasound scan remains the most commonly imaging exam applied to diagnose and assess PPF by Niamey Protocol for the routine evaluation of patients with HSS [13]. It can also reveal signs of portal hypertension, such as splenomegaly, dilation of the portal and splenic veins, and the presence of collateral vessels, which are indicators of variceal formation [14–16].

Indeed, studies involving HSS patients revealed an association between advanced PPF patterns (Niamey) and increased portal vein diameter, as well as a higher risk of esophageal varices bleeding [17, 18]. Additionally, an association has been observed between advanced PPF patterns and the presence of congestive gastropathy [6].

In recent years, liver and spleen stiffness measurement (LSM and SSM) have also been used in the assessment of chronic liver disease and both cirrhotic and non-cirrhotic portal hypertension (NCPH) [19, 20]. Unlike conventional ultrasound, elastography provides direct numerical measurements, making it less operator-dependent and more objective [21]. However, despite this methodological advantage, the widespread deployment of such advanced equipment in remote endemic regions may still be limited. This characteristic allows for safer, faster, and more reproducible assessments of liver and spleen stiffness. Among the available techniques, point shear wave elastography (pSWE) has been applied to patients with schistosomiasis for the evaluation of the liver, spleen, and signs of portal hypertension [22, 23]. Its standardized nature and ease of use are especially advantageous in remote or endemic regions for schistosomiasis, where access to trained personnel and diagnostic infrastructure is often limited [23].

In a consensus on LSM by shear wave elastography, shear wave velocity thresholds were established, with values above 1.7 m/s suggesting compensated advanced chronic liver disease (cACLD) and values above 2.4 m/s indicating clinically significant portal hypertension (CSPH) [19]. CSPH may be associated with the presence of varices and bleeding [24].

Some studies using transient elastography (TE) in hepatosplenic schistosomiasis have demonstrated promising results, particularly with splenic stiffness [25–27]. Others, however, have reported limited sensitivity of TE in detecting clinically relevant outcomes such as periportal fibrosis or variceal bleeding [28]. These contrasting findings highlight the need for further investigation into alternative non-invasive modalities. In this context, point shear wave elastography (pSWE) emerges as a potentially more precise tool, offering objective measurements that may enhance diagnostic accuracy in schistosomiasis-related portal hypertension.

Point shear wave elastography (pSWE) has been applied to patients with schistosomiasis for the evaluation of the liver, spleen, and signs of portal hypertension [22, 23].

Portal hypertension, by promoting splenic congestion, increases spleen stiffness. Due to this pathophysiological relationship, it is acknowledged that SSM has greater accuracy in assessing portal pressure than LSM [29, 30]. However, data on SSM, even in healthy individuals, are not yet well-defined and validated, although a velocity above 3.64 m/s in patients with cirrhosis suggests the presence of varices [31]. HSS data on SSM by pSWE are even more limited [23].

In this context, the combined use of ultrasonography and elastography offers a promising approach to objectively assess disease severity and signs of CSPH in patients with schistosomiasis [13, 32, 33]. This study aimed to evaluate liver disease morbidity and signs of portal hypertension in patients with schistosomiasis, with and without a history of UGIB, using ultrasonography and pSWE of the liver and spleen, in order to identify noninvasive predictors of bleeding risk. The findings may support early risk stratification and monitoring in patients with hepatosplenic schistosomiasis—both for those who have already experienced UGIB and those who have not.

## Methods

### Study design

This is an analytical case-control study involving patients with schistosomiasis with and without a history of gastrointestinal bleeding.

### Patients

Patients were selected from the Schistosomiasis Outpatient Clinic of the Gastroenterology Division at the Hospital das Clínicas, Universidade Federal de Pernambuco (UFPE), Brazil.

Patients diagnosed with *Schistosoma mansoni* infection were included based on their clinical history of contact with water in endemic areas, a past finding of eggs in stool and reports of prior treatment with praziquantel, and ultrasonographic findings of PPF.

All patients meeting the inclusion criteria between 12/2018 and 12/2024 were enrolled and completed a pre-designed questionnaire (available in the supplementary material), and were specifically asked about any history of gastrointestinal bleeding through the occurrence of hematemesis or melena. Data from clinical and laboratory examinations were collected from their medical records.

Patients were excluded for the following: prior splenectomy; presence of fatty liver disease; absence/mild PPF (pattern A or B) [13]; cirrhosis; hepatocellular carcinoma; presence of markers of hepatitis B or C virus infection; other liver diseases; history of drug-induced liver injury or alcohol abuse. Additionally, after pSWE, patients were also excluded if their measurements exhibited an interquartile range (IQR) exceeding 15% of the mean value for liver stiffness and 30% for spleen stiffness. These threshold choices are generally accepted as quality criteria, indicating low variability between repeated measurements. In our study, only one patient had an IQR above the acceptable threshold for liver stiffness (0.34) and was therefore excluded from the analysis to ensure data reliability.

### Procedures

**Fig. 1:**
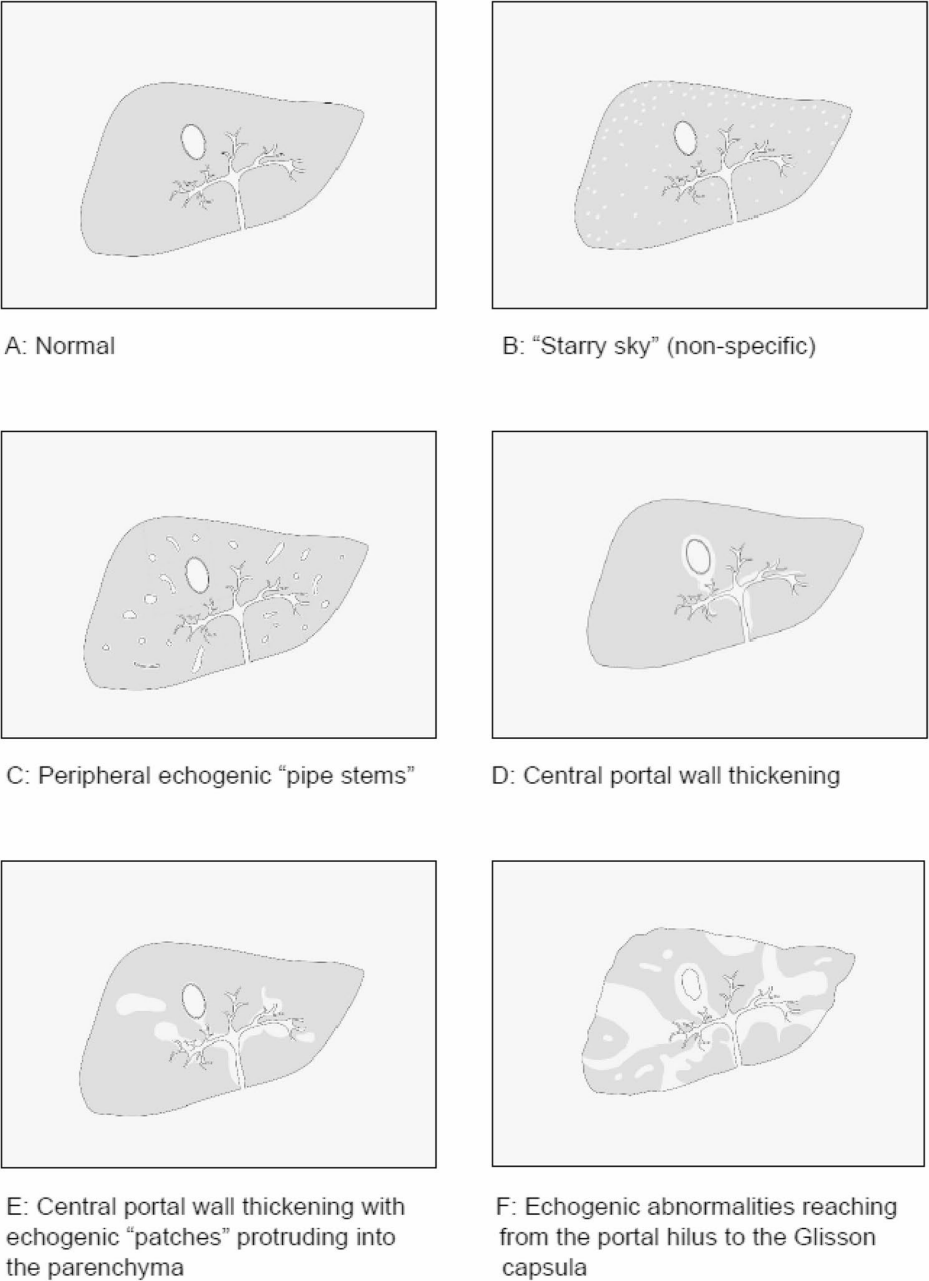
Patterns of Periportal Fibrosis (PPF) according to the Niamey classification

All patients underwent upper abdominal ultrasonography after an 8-hour fasting period, performed by a single experienced operator (ALCD). PPF was classified according to the Niamey protocol, based on the intensity and location of the lesion in the hepatic parenchyma (Fig. 1) [13]. The patterns were defined as C (peripheral fibrosis), D (central fibrosis), E (advanced fibrosis), and F (very advanced fibrosis). The ultrasound examination was conducted using a Siemens Acuson S2000 system (Siemens Medical Solutions, Mountain View, CA, USA) with a 3.5–5 MHz transducer. After ultrasonography scan hepatic and splenic pSWE was performed using this same equipment.

The ultrasonographic signs of portal hypertension assessed included the diameters of the portal vein (PV), splenic vein (SV), longitudinal (spleenL) and transverse (spleenT) diameters of the spleen. Additionally, the splenic index was calculated as spleenL × spleenT, with values up to 60 cm² considered normal [33].

Patients were positioned in the supine position for ultrasonography and pSWE examination, with their right and left arms in maximum abduction to enhance the intercostal acoustic window. Measurements were obtained using the Virtual Touch Quantification (VTQ) software. The region of interest (ROI) was placed for liver stiffness (LS) assessment at least 2.0 cm from the liver capsule, avoiding large hepatic vessels, bile ducts, and fibrotic bands.

Hepatic and splenic pSWE measurements were performed during neutral breathing. Splenic stiffness was assessed in an area free of large vessels, 1–2 cm from the capsule. For each patient, 10 valid shear wave speed (SWS) measurements were obtained, and the results were expressed as the median value of all measurements (m/s). Reliable results were defined as those with an interquartile range (IQR) below 15% for liver measurements and below 30% for spleen measurements, with a success rate of at least 60% [34].

### Statistical analysis

Statistical analysis was performed using SPSS Statistics software, version 26 for Windows (IBM Corporation, Armonk, NY, USA) and MedCalc, version 20.104 (MedCalc Software Ltd., Ostend, Belgium). Initially, the normality of the data distribution was assessed using the Shapiro-Wilk test, which indicated a non-parametric distribution of the quantitative variables analyzed (age, pSWE, spleen size, and portal and splenic vein diameters).

Subsequently, a descriptive analysis of frequency, median, and interquartile range (IQR) was conducted, and patients were stratified into two groups based on the presence or absence of UGIB (upper gastrointestinal bleeding). The chi-square test and Fisher’s exact test were used, depending on the number of observations in each cell, to investigate differences in observed frequencies between the groups regarding sex, Niamey classification and clinical form. The clinical forms of schistosomiasis analyzed in this study are: hepatointestinal, hepatic, and hepatosplenic. The hepatointestinal form is the most frequent, asymptomatic with normal abdominal physical evaluation. In the hepatic form, egg deposition in terminal branches of the portal vein induces granulomas and periportal fibrosis (Symmers’ fibrosis) with hepatomegaly. As this fibrosis progresses, the hepatosplenic form may develop, characterized by hepato/splenomegaly and pre-sinusoidal portal hypertension [2].

To compare the medians of non-parametric variables, the Mann-Whitney test was applied and a binary logistic regression analysis was performed to evaluate the independent variables associated with UGIB. Spearman’s test was used to analyze correlations, as all numerical variables showed a non-parametric distribution. Additionally, after constructing a receiver operating characteristic (ROC) curve and evaluating the area under the curve (AUC), the most appropriate cutoff points were selected to identify patients with schistosomiasis with and without a history of UGIB.

## Results

A total of 177 patients were included the median age was 55 years (IQR: 41–64, range 19–79 years). Of these, 91 were women (51.4%). According to the Niamey classification, 35 patients (19.8%) had peripheral or mild fibrosis (pattern C), 36 patients (20.3%) had central or moderate fibrosis (pattern D), and 106 patients (59.9%) had advanced or very advanced fibrosis (patterns E and F).

In Table 1 are described the demographic and clinical characteristics, ultrasonography parameters, LSM and SSM values measured by pSWE in the 177 patients with schistosomiasis, with and without a history of UGIB.

**Table 1:** Parameters of 177 patients with schistosomiasis with/without UGIB history, pernambuco, Brazil, 2024

Characteristics	Total (*n* = 177)		UGIB				*P*
			Present (*n* = 51)		Absent (*n* = 126)		
Sex (n,%)							
Male	86	(48.6%)	25	(49.0%)	61	(48.4%)	0.942^a^
Female	91	(51.4%)	26	(51.0%)	65	(51.6%)	
Age (years)*	55	(41–64)	53	(42–64)	56	(40–64%)	0.708^b^
Clinical form (n,%)							
HIS	39	(22.0%)	0	(0.0%)	39	(31.0%)	**<0.001** ^**a**^
HS	25	(14.1%)	0	(0.0%)	25	(19.8%)	**< 0.001** ^**c1**^
HSS	113	(63.8%)	51	(100.0%)	62	(49.2%)	**< 0.001** ^**c2**^
Niamey classification (n,%)							
C	35	(19.8%)	0	(0.0%)	35	(27.8%)	**0.001** ^**a**^
D (D/DC)	36	(20.3%)	8	(15.7%)	28	(22.2%)	**0.005** ^**c3**^
E + F (E/EF/F)	106	(59.9%)	43	(84.3%)	63	(50.0%)	**< 0.001** ^**c4**^
Portal vein diameter* (cm)	1.10	0.94–1.32)	1.16	(1.01–1.40)	1.07	(0.89–1.26)	**0.034** ^**b**^
Splenic vein diameter* (cm)	0.79	(0.57–1.01)	0.98	(0.82–1.22)	0.69	(0.54–0.94)	**< 0.001** ^**b**^
Spleen longitudinal diameter* (cm)	13.8	(10.6–17.0)	16.6	(14.7–19.1)	12.2	(10.0-15.6)	**< 0.001** ^**b**^
Spleen transverse diameter* (cm)	5.3	(4.07–6.61)	6.5	(5.88–7.72)	4.6	(3.8–5.9)	**< 0.001** ^**b**^
Splenic Index	75.1	(42.9-109.4)	105.8	(83.0-137.2)	56.5	(38.4–93.7)	**< 0.001** ^**b**^
pSWE Hepatic*							
Median (m/s)	1.38	(1.17–1.65)	1.54	(1.27–1.89)	1.34	(1.16–1.57)	**0.004** ^**b**^
IQR**	0.14	(0.09–0.19)	0.16	(0.10–0.23)	0.13	(0.08–0.18)	0.101^b^
pSWE Splenic*							
Median (m/s)	3.42	(2.81–3.77)	3.65	(3.29–3.90)	3.28	(2.58–3.73)	**< 0.001** ^**b**^
IQR**	0.33	(0.23–0.47)	0.33	(0.21–0.42)	0.34	(0.25–0.48)	0.337^b^

*HIS* Hepatointestinal schistosomiasis, *HS* Hepatic schistosomiasis, *HSS* Hepatoesplenic schistosomiasis, *IQR* Interquartile range, *pSWE* Point shear wave elastography, *UGIB* Upper gastrointestinal bleeding

*Values expressed as median (IQR), with the Shapiro-Wilk normality test < 0.05 indicating a non-parametric distribution of the variables

^a^Chi-square test; ^b^Mann-Whitney test; ^c^Fisher’s exact test

^c1^p-value of Fisher’s exact test comparing the HS and HSS clinical forms among patients with and without UGIB;

^c2^p-value of Fisher’s exact test comparing the HIS and HSS clinical forms among patients with and without UGIB. The comparison between the HIS and hepatic clinical forms among patients with and without UGIB was not statistically significant (*p* = 1.000)

^c3^p-value of Fisher’s exact test comparing the Niamey patterns “C” and “D”; ^c4^p-value of Fisher’s exact test comparing the Niamey classifications “C” and “E + F”. The comparison between the Niamey classifications “D” and “E + F” was not statistically significant (*p* = 0.424)

**IQR comparison between patients with and without UGIB

According to Table 1, a highly significant difference between the UGIB and the non-bleeding group was observed. The UGIB group showed a higher frequency of the hepatosplenic form (*p* < 0.001) and a more advanced PPF pattern (E + F, 84.3% vs. 50%, *p* < 0.001). Morphologically, the UGIB group also had larger diameters of the portal vein (1.16 cm vs. 1.07 cm; *p* = 0.034) and splenic vein (0.98 cm vs. 0.69 cm; *p* < 0.001). Spleen dimensions were also greater in the UGIB group, including the longitudinal diameter (16.6 vs. 12.2 cm; *p* < 0.001), transverse diameter (6.5 vs. 4.6 cm; *p* < 0.001), and the resulting splenic index (105.8 vs. 56.5; *p* < 0.001). Furthermore, both hepatic and splenic stiffness, measured by pSWE, were significantly higher in the UGIB group (1.54 vs. 1.34 m/s, *p* = 0.004; and 3.65 vs. 3.28 m/s, *p* < 0.001, respectively).

Based on the binary logistic regression analysis, the only variable considered as an independent predictor of UGIB in this model was the “splenic longitudinal diameter”, as shown on Table 2. This means that for every centimeter increase in the longitudinal diameter of the spleen, the odds of a patient having a history of upper gastrointestinal bleeding increase by a factor of 2.214, considering all other variables in the model constant.

**Table 2:** Binary logistic regression analysis of factors associated with upper gastrointestinal bleeding (UGIB) in patients with schistosomiasis, pernambuco, Brazil, 2024

Variáveis	B (Coefficient)	Standard Error	*p*-value	OR	(95% CI)
Niamey Classification			0,717		
C (vs. E + F)	0,838	13572,59	1	2,311	(0.000,)
D (vs. E + F)	0,493	0,604	0,414	1,637	(0.501, 5.342)
Clinical form			1		
HIS (vs. HSS)	−18,969	12758,8	0,999	0	(0.000,)
HS (vs. HSS)	−19,332	7562,732	0,998	0	(0.000,)
Portal vein diameter (cm)	−0,82	0,701	0,242	0,44	(0.112–1.738)
Splenic vein diameter (cm)	−0,037	0,794	0,963	0,963	(0.203–4.569)
Spleen longitudinal diameter (cm)	**0**,**795**	**0**,**379**	**0**,**036**	**2**,**214**	**(1.053–4.656)**
Spleen transverse diameter (cm)	1,581	0,927	0,088	4,86	(0.791–29.882)
Splenic Index	−0,093	0,054	0,084	0,911	(0.819–1.012)
Hepatic pSWE (m/s)	0,487	0,352	0,166	1,627	(0.817–3.242)
Slpenic pSWE (m/s)	0,236	0,392	0,547	1,267	(0.587–2.731)

*CI* Confidence interval, *HIS* Hepatointestinal schistosomiasis, *HS* Hepatic schistosomiasis, *HSS* Hepatoesplenic schistosomiasis, *OR* Odds ratio, *pSWE* Point shear wave elastography, *UGIB* Upper gastrointestinal bleeding

**Fig. 2:**
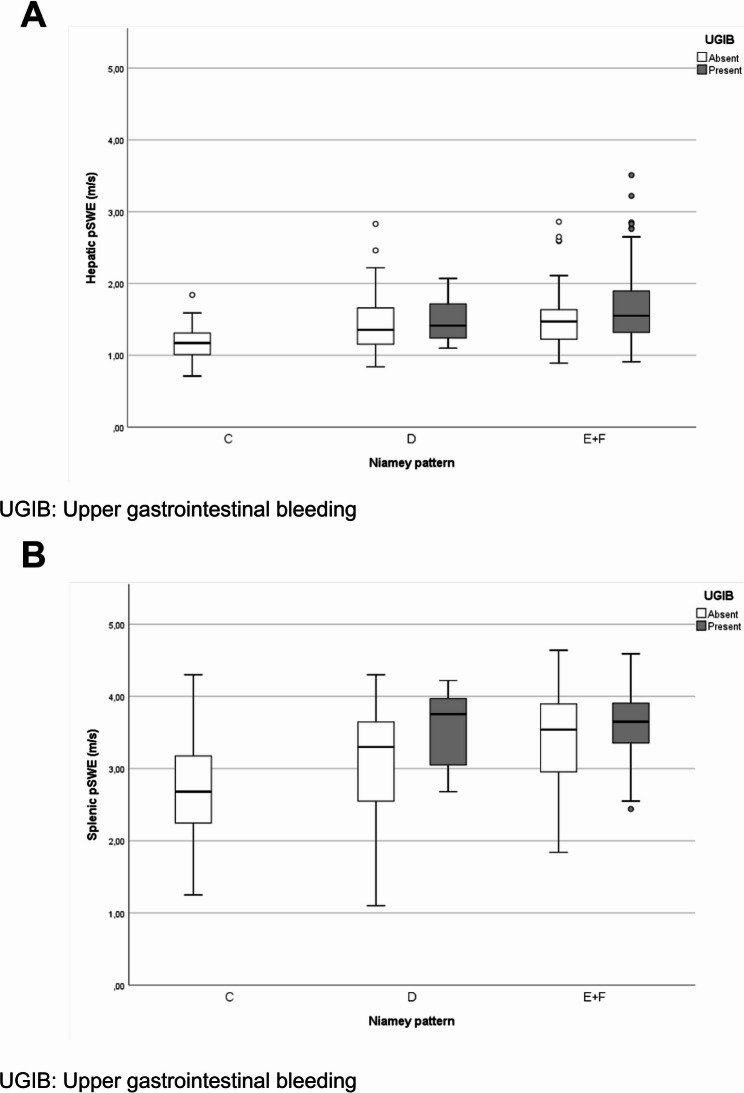
Hepatic (**A**) and splenic (**B**) pSWE, according to PPF pattern based on the Niamey classification

Figure 2 shows the median acoustic wave velocity (m/s) obtained from hepatic (A) and splenic (B) elastography, according to the PPF pattern based on the Niamey classification, in 177 patients with schistosomiasis, with and without a history of UGIB. Despite, as known [35], the median wave velocity in spleen are higher than liver, there were no significant differences. However, there were no significant differences in wave velocity between patients with and without UGIB within each PPF pattern group.

**Fig. 3:**
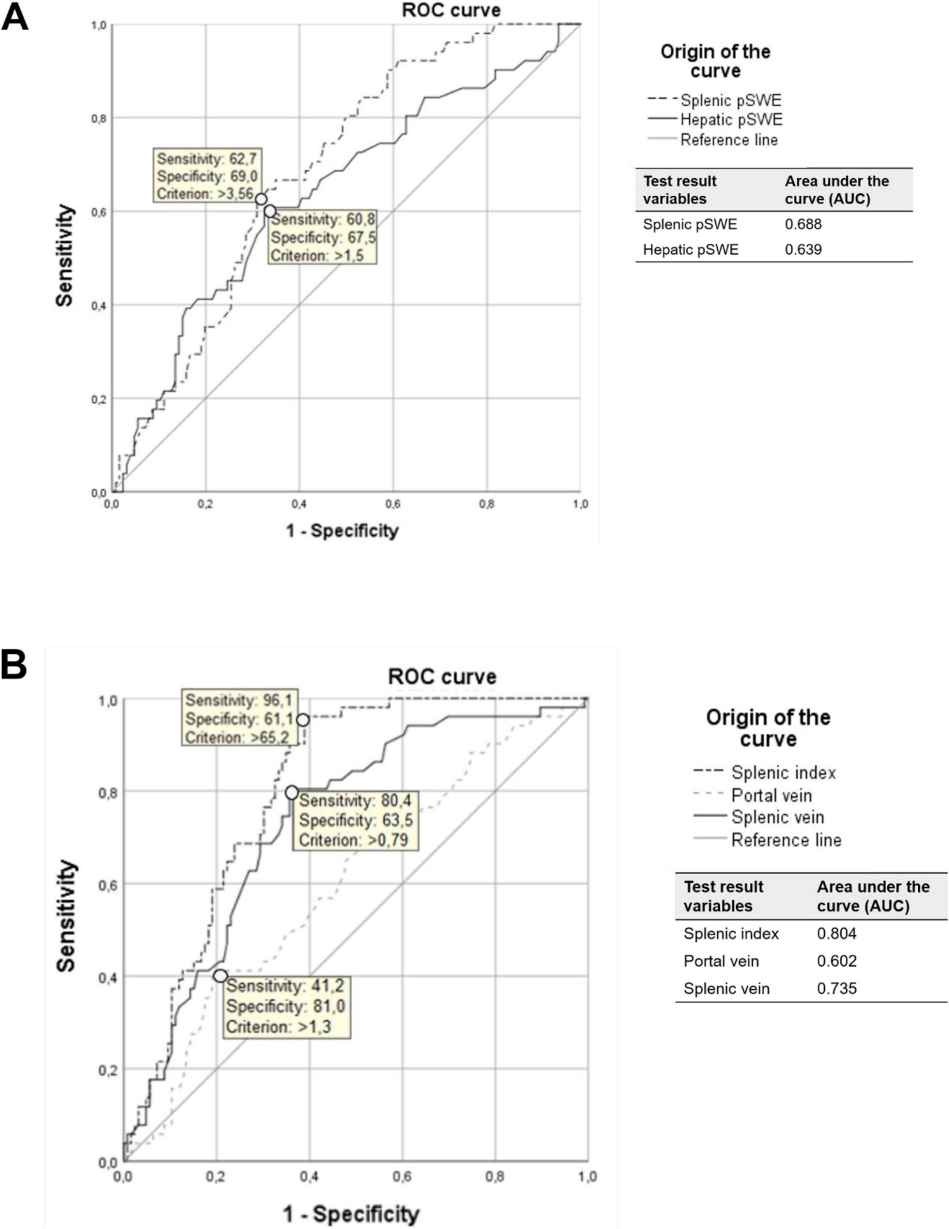
ROC curve of hepatic (**A**) and splenic (**B**) pSWE differentiating patients with schistosomiasis by UGIB history

Figure 3 presents the ROC curve of the acoustic wave velocities (m/s) obtained from hepatic and splenic elastography (1 A) and the splenic index, portal vein diameter, and splenic vein diameter (1B), with the best cutoff points to differentiate the 177 patients with schistosomiasis, with and without a history of UGIB.

Table 2 describes the sensitivity, specificity, positive likelihood ratio, negative likelihood ratio, and accuracy of hepatic and splenic pSWE, splenic index, portal vein diameter, and splenic vein diameter and Niamey’s PPF patterns to identify the 177 patients with schistosomiasis, with and without a history of UGIB.

ROC curve analysis was performed to distinguish patients with a history of UGIB. The optimal cut-off values with their corresponding sensitivity (Se) and specificity (Sp) were: portal vein diameter > 1.3 cm (Se = 41.2%, Sp = 81.0%), splenic vein diameter > 0.79 cm (Se = 80.4%, Sp = 63.5%), splenic index > 65.2 (Se = 96.1%, Sp = 61.1%), liver pSWE velocity > 1.5 m/s (Se = 60.8%, Sp = 67.5%), and spleen pSWE velocity > 3.56 m/s (Se = 62.7%, Sp = 69.0%), as shown in Fig. 3. Among these variables, the splenic index provided the best discriminatory performance (AUC = 0.804). Its high sensitivity (96.1%) suggests it is an excellent screening tool to identify high-risk patients; however, its modest specificity (61.1%) limits its value for confirming a positive finding.

According to Table 3, most positive likelihood ratios (LR+) were above 2.0. This indicates that findings such as splenic pSWE > 3.56, splenic index > 65.2, portal vein diameter > 1.3 cm, and splenic vein diameter > 2.2 cm were associated with a small to moderate increase in the probability of UGIB. In contrast, the splenic index demonstrated robust performance in ruling out the condition, with a negative likelihood ratio (LR-) of 0.064. This finding implies that a splenic index < 65.2 is a strong predictor for the absence of UGIB.

**Table 3:** Sensitivity, specificity and LR+, LR-, and accuracy of hepatic and splenic pSWE, spleen and vein diameters

	UGIB					Sensitivity		Specificity		LR+		LR-		A
	Yes *n* = 51		No *n* = 126		p*	(%)	IC95%	(%)	IC95%	(%)	IC95%	(%)	IC95%	(%)
Splenic pSWE	n	%	n	%										
> 3.56 (*n* = 71)	32	62.7	39	31.0	< 0.001	62.7	48.1–75.9	69.1	60.2–77.0	2.03	1.45–2.84	0.54	0.37–0.78	**67.2**
≤ 3.56 (*n* = 106)	19	37.3	87	69.0										
Hepatic pSWE	n	%	n	%										
> 1.5 (*n* = 72)	31	60.8	41	32.5	0.003	60.8	46.1–74.2	67.5	58.5–75.5	1.87	1.34–2.61	0.58	0.40–0.84	**65.5**
≤ 1.5 (*n* = 105)	20	39.2	85	67.5										
Splenic Index	n	%	n	%										
> 65.2 (*n* = 99)	49	96.1	50	39.7	< 0.001	96.1	86.5–99.5	61.1	52.0-69.7	2.47	1.97–3.10	0.064	0.016–0.25	**70.6**
≤ 65.2 (*n* = 78)	2	3.9	76	60.3										
Portal vein	n	%	n	%										
> 1.3 (*n* = 45)	21	41.2	24	19.0	0.029	41.2	27.6–55.8	80.9	73.0-87.4	2.16	1.33–3.52	0.73	0.57–0.93	**69.5**
≤ 1.3 (*n* = 132)	30	58.8	102	81.0										
Splenic vein	n	%	n	%										
> 0.79 (*n* = 87)	41	80.4	46	36.5	< 0.001	80.4	66.9–90.2	63.5	54.4–71.9	2.20	1.69–2.88	0.31	0.17–0.55	**68.4**
≤ 0.79 (*n* = 90)	10	19.6	80	63.5										
Niamey’s PPF patterns	n	%	n	%										
C + D (*n* = 71)	8	15.7	63	50.0	< 0.001	84.3	71.4–93.0	50	41.0–59.0	1.69	1.37–2.08	0.31	0.16–0.61	**59.9**
E + F (*n* = 106)	43	84.3	63	50.0										

*LR+ *Positive Likelihood Ratio, *LR-* Negative Likelihood Ratio, *A* Accuracy, *pSWE* Point shear wave elastography

*Chi-square test

## Discussion

Digestive bleeding caused by the rupture of varices constitutes the most severe condition in *S. mansoni* infection, as it can trigger decompensation of liver disease and lead to death [9, 11].

In this study, among the 177 patients with *Schistosoma mansoni* infection and PPF pattern C or higher, 51 (28.8%) had a history of UGIB. Among them, the majority (84.3%) had advanced PPF (pattern E or F), which has been associated with greater splenic enlargement and increased likelihood of portal hypertension [36].

In addition, findings such as a splenic pSWE > 3.56, splenic index > 65.2, portal vein diameter > 1.3 cm, and splenic vein diameter > 2.2 cm were approximately twice as likely to be present in patients with UGIB compared to those without this complication. Conversely, a splenic index ≤ 65.2 was a strong indicator for the absence of UGIB, as this finding was nearly 15 times more common in patients without UGIB than in those with it. Moreover, a splenic vein diameter ≤ 0.79 cm and C-D Niamey’s PPF patterns were also predictive of the absence of UGIB, being about three times more frequent in patients without the complication.

The association between advanced PPF patterns and clinical outcomes, such as the degree of esophageal varices and UGIB, has been well-documented [16, 17, 37]. In a large multicenter study involving nearly 4,000 HSS patients from Egypt and Kenya, advanced PPF was correlated with increased portal vein diameter, thickening of the portal branch walls, and a higher risk of bleeding [16]. Our findings further support this association, as patients with UGIB history exhibited significantly larger portal and splenic vein diameters, as well as higher splenic indices, compared to those without UGIB antecedent.

Additionally, the splenic index in patients with schistosomiasis is a useful and easily measurable Marker by ultrasonography. A study involving 58 patients with schistosomiasis revealed that a splenic index above 144 was indicative of rebleeding, with high accuracy [30]. In the present study, it was observed that the splenic index was the parameter with the best accuracy (AUC = 0.804) in identifying patients with schistosomiasis with a history of UGIB, demonstrating better performance than portal vein diameter or splenic vein diameter or Niamey’s PPF patterns, as well as outperforming hepatic and splenic pSWE.

In contrast to what was observed here for schistosomiasis, hepatic and splenic pSWE constitute an important tool in the evaluation of portal hypertension in patients with cACLD, as it can also suggest CSPH and varices [17, 38, 39]. Specifically, data from the literature have described that SSM demonstrates better accuracy in assessing both cirrhotic and non-cirrhotic portal hypertension and in predicting varices than LSM [28, 40]. Still, some studies suggest using LSM, SSM, and platelet count to confirm the diagnosis of CSPH and the presence of varices [17, 19, 25, 41]. This difference may be explained by the distinct pathophysiological mechanisms of periportal fibrosis in schistosomiasis, which predominantly affects the portal tracts while preserving most of the hepatic parenchyma, compared with the diffuse architectural distortion and parenchymal dysfunction observed in cirrhosis.

Although data on hepatic and splenic elastography remain scarce in the evaluation of morbidity and NCPH in schistosomiasis, some results appear promising [19–21, 42].

In 2017, it was observed in 30 patients with schistosomiasis, 30 patients with cirrhosis with HCV and 17 controls, that transient elastography of the liver showed higher velocity in patients with cirrhosis than in those schistosomotic [23]. Another study revealed that liver wave velocity measured by pSWE was higher in patients with schistosomiasis with more advanced PPF (Niamey D/E/F) than in those with milder PPF (Niamey C) [20].

A study using transient elastography, involving cirrhotic and patients with schistosomiasis, revealed that liver wave velocity (kPa) was twice as high in patients with cirrhosis. On the other hand, in patients with schistosomiasis, spleen wave velocity was higher than in patients with cirrhosis, suggesting transient elastography as a tool for the differential diagnosis between these two liver diseases. A cutoff point > 11.75 kPa for liver wave velocity (AUC = 0.947) suggested a diagnosis of cirrhosis [42].

In the present study, the cutoff point > 1.5 m/s for hepatic pSWE, which distinguishes patients with schistosomiasis with a history of UGIB, was lower than that suggesting CSPH (> 2.4 m/s) in patients with cirrhosis [17]. This could be explained by the fact that, as in schistosomiasis the fibrosis does not involve the hepatic parenchyma diffusely, but is localized around the portal branches (PPF), it may result in lower liver stiffness compared to cirrhosis. Moreover, in portal hypertension due to HSS, additionally to the portal obstructive process caused by egg deposition, there is also greater splenic congestion due to primary splenomegaly, explaining the finding of higher spleen stiffness compared to patients with cirrhosis [42]. In fact, a recent study described an association between splenic pSWE wave velocity and PPF patterns, i.e., the splenic higher velocity and more advanced PPF pattern, but interestingly this was not observed with hepatic pSWE [21].

In this study involving only patients with schistosomiasis, the wave velocities measured by pSWE, both in the liver and spleen, were higher in those with a history of UGIB, reflecting greater visceral stiffness and possibly higher pressure levels in the portal territory. These findings indirectly suggest that pSWE has the potential to be used in combination with other parameters, such as the splenic index, in the screening of patients with schistosomiasis at bleeding risk.

Splenic longitudinal diameter was also considered in the multivariate analysis as a powerful indicator of advanced disease and bleeding risk. This finding aligns with the prior univariate analysis and the ROC curve results, The lack of significance for the other variables, particularly the Niamey classification and the pSWE measures, suggests a potential issue within the model, such as multicollinearity. The high correlation between different measures of spleen size (longitudinal diameter, transverse diameter, and spleen index) could have caused the model to only find one of them to be significant while suppressing the others.

Tissue elasticity varies according to organ structure and is altered in disease states. Although spleen stiffness is inherently higher than liver stiffness [42], our findings showed that higher median pSWE values (m/s) among patients with UGIB was marginal and not statistically significant.

A recent study involving 51 patients with HSS, using ultrasonography and transient elastography of the liver and spleen to predict the presence of esophageal varices, revealed that splenic parameters performed better. The authors concluded that splenic vein diameter and SSM are useful tools for predicting esophageal varices in patients with HSS [25]. Indeed, in the present study, ultrasonographic parameters related to the spleen, particularly the splenic index, as well as SSM, were also more accurate in identifying patients with schistosomiasis with a history of UGIB.

A major limitation of our study was the absence of endoscopic evaluation for the diagnosis of esophageal varices. The reliance on a convenience sampling method represents an additional constraint on the interpretation of the results. Nevertheless, this was the first prospective study involving patients with schistosomiasis, using ultrasonography and hepatic and splenic pSWE, in which the parameter used for the diagnosis of CSPH was a history of UGIB reported by the patient. It is known, however, that since this is an indirect measure, reliance on patient-reported history of blood loss (hematemesis or melena) introduces potential recall bias, especially in cases of melena, where the bleeding is less noticeable and may be underestimated or misinterpreted by the patient.

## Conclusion

Spleen-related ultrasonographic and elastographic parameters, particularly splenic index, proved to be promising tools for identifying patients with schistosomiasis with a history of UGIB. Further studies involving a larger number of patients, using endoscopy as the parameter for diagnosing varices and predictive factors of bleeding, will be necessary to corroborate these findings.

## Supplementary Information

Supplementary Material 1

## Data Availability

The datasets used and/or analysed during the current study are available from the corresponding author on reasonable request.

## Abbreviations

AUC: Area under the curve
CA: California
cACLD: Compensated advanced chronic liver disease
CSPH: Clinically significant portal hypertension
IQR: Interquartile Range
HIS: Hepatointestinal schistosomiasis
HS: Hepatic schistosomiasis
HSS: Hepatosplenic schistosomiasis
kPa: Kilopascals
Ltd: Limited
LR+: Positive Likelihood Ratio
LR-: Negative Likelihood Ratio
LSM: Liver stiffness measurement
NCPH: Non-cirrhotic portal hypertension
NY: New York
PPF: Periportal fibrosis
pSWE: Point shear wave elastography
PV: Portal vein
ROC: Receiver operating characteristic
Se: Sensitivity
Sp: Specificity
spleenL: Longitudinal diameter of the spleen
spleenT: Transverse diameter of the spleen
SSM: Spleen stiffness measurement
SV: Splenic vein
UGIB: Upper gastrointestinal bleeding
USA: United States of America
