# Supplementary material for: Liver and spleen ultrasonography and elastography are useful for identifying a history of upper gastrointestinal bleeding in patients with schistosomiasis
Source: BMC Infect Dis. 2026 Jan 29;26:268. doi: 10.1186/s12879-025-11635-6 (PMC12875039; doi:10.1186/s12879-025-11635-6)
Supplement: Supplementary file 1 — Supplementary Material 1 [file 12879_2025_11635_MOESM1_ESM.pdf]

SCHISTOSOMIASIS QUESTIONNAIRE - Hospital das Clínicas / Federal University of Pernambuco	
MEDICAL RECORD:	
FULL NAME:	
PLACE OF BIRTH / NATIONALITY:	
CITY OF RESIDENCE / STATE-PROVINCE:	
SEX / GENDER:	
AGE:	
DATE OF BIRTH:	
PHONE NUMBER:	
WEIGHT (kg):	
HEIGHT (cm):	
DIABETIC PATIENT:	*NO ( ) YES ( ) If yes, please specify current medication:*
LAST CONTACT WITH RIVER WATER:	*NO ( ) YES ( ) If yes, specify location:*
PRIOR SCHISTOSOMIASIS TREATMENT:	*NO ( ) YES ( ) If yes, indicate treatment date(s):*
HEMATEMESIS:	*NO ( ) YES ( ) If yes, specify frequency and most recent episode:*
MELENA:	*NO ( ) YES ( ) If yes, specify frequency and most recent episode:*
ALCOHOL USE DISORDER:	*NO ( ) YES ( ) If yes, specify quantity and frequency:*
HEPATITIS:	*NO ( ) YES ( ) Type; Date of diagnosis; Treatment:*
SUBSTANCE USE :	*NO ( ) YES ( ) If yes, specify substance(s) and frequency:*
FAMILY HISTORY OF SCHISTOSOMIASIS:	*NO ( ) YES ( ) Number of affected relatives; Relationship(s):*
